# The place of hyperbaric oxygen therapy and ozone therapy in sudden hearing loss^☆^

**DOI:** 10.1016/j.bjorl.2016.06.002

**Published:** 2016-07-06

**Authors:** Gülin Ergun Taşdöven, Alper Tunga Derin, Neslihan Yaprak, Hasan Ümit Özçağlar

**Affiliations:** a Van Training And Research Hospital, Department of Otorhinolaryngology, Van, Turkey; b Akdeniz University, School of Medicine, Department of Otorhinolaryngology, Antalya, Turkey

**Keywords:** Hyperbaric oxygen therapy, Idiopathic sudden sensorineural hearing loss, Oral steroid, Ozone therapy, Oxigenoterapia hiperbárica, Surdez súbita, Esteroide oral, Ozonioterapia

## Abstract

**Introduction:**

It is difficult to evaluate the effect of drugs clinically used for idiopathic sudden sensorineural hearing loss, mainly because its underlying mechanism remains unknown.

**Objective:**

This study assessed the efficacy of hyperbaric oxygen therapy or ozone therapy in the treatment of idiopathic sudden sensorineural hearing loss, when either therapy was included with steroid treatment.

**Methods:**

A retrospective analysis examined 106 patients with idiopathic sudden sensorineural hearing loss seen between January 2010 and June 2012. Those with an identified etiology were excluded. The patients were divided into three treatment groups: oral steroid only (*n* = 65), oral steroid + hyperbaric oxygen (*n* = 26), and oral steroid + ozone (*n* = 17). Treatment success was assessed using Siegel criteria and mean gains using pre- and post-treatment audiograms.

**Results:**

The highest response rate to treatment was observed in the oral steroid + ozone therapy group (82.4%), followed by the oral steroid + hyperbaric oxygen (61.5%), and oral steroid groups (50.8%). There were no significant differences in the response to treatment between the oral steroid and oral steroid + hyperbaric oxygen groups (*p* < 0.355). The oral steroid + ozone group showed a significantly higher response rate to treatment than the oral steroid group (*p* = 0.019). There were no significant differences between the oral steroid + hyperbaric oxygen and oral steroid + ozone groups (*p* = 0.146).

**Conclusion:**

The efficiency of steroid treatment in patients with severe hearing loss was low. It was statistically ascertained that adding hyperbaric oxygen or ozone therapy to the treatment contributed significantly to treatment success.

## Resumo

**Introdução:**

É difícil avaliar o efeito dos fármacos clinicamente utilizados na surdez súbita idiopática, principalmente porque o seu mecanismo subjacente se mantém desconhecido.

**Objetivo:**

Avaliar a eficácia da oxigenoterapia hiperbárica ou ozonioterapia no tratamento de surdez súbita, quando uma ou outra terapia é incluída no tratamento com esteroides.

**Método:**

Uma análise retrospectiva examinou 106 pacientes com surdez súbita atendidos entre janeiro de 2010 e junho de 2012. Aqueles com uma etiologia identificada foram excluídos. Os pacientes foram divididos em três grupos de tratamento: apenas esteroide oral (n = 65), esteroide por via oral + oxigenoterapia hiperbárica (n = 26) e esteroides por via oral + ozônio (n = 17). O sucesso do tratamento foi avaliado com critérios de Siegel e os ganhos médios com audiogramas pré e pós-tratamento.

**Resultados:**

A taxa de resposta mais elevada para o tratamento foi observada no grupo de esteroide + ozonioterapia (82,4%), seguida por grupos de esteroide oral + oxigenoterapia hiperbárica (61,5%) e esteroide oral (50,8%). Não houve diferenças significantes na resposta ao tratamento entre os grupos de esteroide oral e esteroides + oxigenoterapia hiperbárica (*p* < 0,355). O grupo de esteroide oral + ozônio apresentou uma taxa de resposta significantemente mais elevada ao tratamento do que o grupo de esteroide oral (*p* = 0,019). Não houve diferenças significantes entre os grupos de esteroide oral + oxigenoterapia hiperbárica e esteroide oral + ozônio (*p* = 0,146).

**Conclusão:**

A eficiência do tratamento com esteroides em pacientes com perda auditiva grave foi baixa. Verificou-se estatisticamente que a adição de oxigenoterapia hiperbárica ou ozonioterapia ao tratamento contribuiu significantemente para o sucesso do tratamento.

## Introduction

Idiopathic sudden sensorineural hearing loss (ISSNHL) is an otologic disease requiring urgent diagnosis and treatment. ISSNHL is commonly defined as hearing loss of more than 30 dB, affecting three or more frequencies, arising over less than 3 days, without an identifiable etiology.^1^ The hearing loss develops within a few seconds, minutes, or hours. In several epidemiological studies conducted on the incidence of ISSNHL, the spontaneous recovery rate of ISSNHL is high; the actual incidence is estimated to be far above this value.

Although several factors account for its etiology, most cases are idiopathic. Although there are more than 100 considered etiologic causes, the widely accepted etiological theories are viral infections, vascular causes, cochlear membrane disorders, and autoimmunity. In many cases, however, no apparent cause can be indicated.^2^, ^3^, ^4^ The etiology can be clarified in 10–15% of cases, with ISSNHL diagnosed in the remainder.^2^, ^4^, ^5^

Although ISSNHL recurs spontaneously in 32–65% of cases,^6^ the reported rate ranged between 49% and 89% when steroids were used during treatment.^7^ Steroids remain the most commonly used medication for the treatment of ISSNHL.

The idea that ISSNHL could occur due to hypoxia in the cochlear apparatus makes hyperbaric oxygen therapy (HBOT) a reasonable choice. Generally, HBOT is recommended for the treatment of ISSNHL as a supplementary therapy to the first-line medical treatment.

Recently, ozone therapy has been used as a supplementary treatment for diseases where inflammatory processes are preponderant and an ischemic etiology is found. Ozone therapy is considered a treatment for ISSNHL because its effects, such as enhancing oxygen, glucose, and adenosine triphosphate (ATP) delivery to ischemic tissues, in turn producing reactive oxygen derivatives, result in vasodilation by increasing the amount of nitric oxide, stimulating angiogenesis and providing immunomodulation.

Our study included 106 patients with ISSNHL who were treated at our clinic between 2010 and 2012. We retrospectively investigated the efficacy of the treatment protocols applied to patients with ISSNHL, and studied the role of HBOT or ozone therapy for the treatment of ISSNHL with either therapy administered as a supplement to systemic steroid treatment.

## Methods

### Patients

A retrospective chart review was performed 106 patients with a diagnosis of ISSNHL who presented at the Department of Otorhinolaryngology – Head and Neck Surgery at Akdeniz University, between January 2010 and June 2012. The inclusion criteria were the same for each group of treatment. The inclusion criteria were unilateral sensorineural hearing loss with an average hearing loss of 30 dB in consecutive frequencies developing within 3 days. Pediatric patients, patients with preexisting Meniere's disease, tumors, acoustic trauma, barotrauma, retrocochlear disease, bilateral hearing loss, those with a history of chronic otitis in the same ear, and those with a history of surgery of the same ear were not included in the study. Those patients with an identified cause were excluded, and only idiopathic cases were investigated. Bilateral cases were also not included. In all, 62 (58.5%) of the study participants were male and 44 (41.5%) were female. The mean age was 50 ± 13 years; the oldest person was 81 years, whereas the youngest person was 17 years. All of the patients had repeated audiological tests on the first, third, fifth, seventh, and fifteenth days of treatment. In accordance with the American Speech and Hearing Association guidelines, hearing loss was classified as mild (26–40 dB), moderate (41–55 dB), moderate-severe (56–70 dB), severe (71–90 dB), and profound or total (91 dB and above) loss according to the average pure tone thresholds at 500, 1000, 2000, and 4000 Hz. Temporal magnetic resonance imaging was requested for all of the patients to evaluate the acoustic channel and brain stem.

### Treatment groups

The patients were divided into three groups according to the treatment protocol received: oral steroid (Group A), oral steroid + HBOT (Group B), and oral steroid + ozone (Group C). The oral steroid group comprised 63 patients, the oral steroid + HBOT group comprised 26 patients, and the oral steroid + ozone group comprised 17 patients. According to our clinic's protocols for ISSNHL with oral steroids, all of the patients were given 1 mg/kg/day oral prednisolone for 5 days, the maximum dose given to patients was 60 mg/day, which was subsequently tapered by 10 mg every 2 days; at the same time, 30 mg/day gastroprotective oral lansoprazole and a salt-free diet combination were administered. The oral steroid treatment is done for all the patients routinely. During the past 6 years, hyperbaric oxygen therapy and ozone therapy has been applied as adjunctive treatment in our clinic for ISSNHL. Since then, we have given information and recommend about the ozone therapy and hyperbaric oxygen treatment. And if the patient want one of these treatments; this treatment add to the steroid treatment. Since the hyperbaric oxygen therapy and ozone therapy which are supplementary treatment methods to the routine treatments, are not paid for by the insurance companies in our country, none of our patients could receive these treatments.

The socio-economic and transportation status was influential in choosing the treatment method. The reason for the number of the patients who received hyperbaric oxygen therapy and ozone therapy being few stems from the fact that not all patients accept this treatment because it has a fee to be paid.

The patients in the oral steroid + HBOT group, in addition to receiving oral steroids according to the ISSNHL protocol, received HBOT applied as 100% oxygen inhalation at 2.5 ATA pressure for 90 min, which was administered once a day within a 10 day period (Hiperbot Model 101; Hiperbot Ltd, İstanbul).

The patients in the oral steroid + ozone group, in addition to receiving oral steroids according to the ISSNHL protocol, received medical ozone therapy using the major autohemotherapy method. In this method, an anti-ozonant infusion set (Ozonosan; Mikro-Perl-System; Dr. J. *Hänsler*, Iffezheim, Germany) and 100 mL blood drawn from the patient were mixed, under sterile conditions, with 99.5% oxygen and 0.5% of an ozone mixture (the ozone concentration within the mixture = 20 μg/mL) obtained from an ozone generator (Medozon Compact; Herrmann Apparatebau, Kleinwallstadt, Germany); this blood mixture was again administered to the patient intravenously, at least for a 5-min period. During the course of the process, sodium citrate was used as an anticoagulant. This therapy was performed in five sessions twice a week. The ozone therapy protocol that used by Ragab and his friends on their search about this topic; also used as source (Ragab A, Shreef E, Behiry E, Zalat S, Noaman M. Randomised, double blinded, placebo-controlled, clinical trial of ozone therapy as treatment of sudden sensorineural hearing loss. J Laryngol Otol. 2009;123:54–60).

### Audiometric investigation

To determine the change in post-treatment hearing, pre-treatment audiological examination and post-treatment audiological examination were performed on the fifteenth day (the threshold averages at 500, 1000, 2000, and 4000 Hz were compared) (Clinical Audiometers AC-40 Interacoustics, Assens, Denmark).

The results were evaluated considering changes in Speech Discrimination Scores (SDSs) and Pure Tone Averages (PTAs) at 500, 1000, 2000, and 4000 Hz.

The response to therapy was categorized according to Siegel's criteria^8^ as follows:

1. Healing: final threshold more than 25 dB.
2. Partial improvement: gain of more than 15 dB, final hearing threshold 25–45 dB.
3. Slight improvement: gain of more than 15 dB, final hearing threshold more than 45 dB.
4. No response: gain of less than 15 dB and final hearing threshold more than 75 dB.

A PTA greater than 15 dB was considered a response to treatment, whereas a PTA less than 15 dB was deemed no response to treatment. If the increase in the pre- vs. post-treatment SDS of the patients was more than 20%, the value was considered a response to treatment; however, if the increase was less than 20%, the value was considered no response to treatment.

The audiograms were classified as upsloping, downsloping, flat, and total deafness according to the hearing thresholds at different frequencies. An upsloping curve was defined as a more severe (>20 dB) hearing loss at low (250 and 500 Hz) frequencies, whereas a downsloping curve was defined as a more severe (>20 dB) hearing loss at high (4000 and 8000 Hz) frequencies. An audiometric curve with no more than 15 dB difference at any frequency was accepted as being flat.

### Statistical analysis

All statistical analyses were performed using the Statistical Package for the Social Sciences (SPSS) for Windows 7.0 PASW Statistics 18 (SPSS, Chicago, IL, USA). A two-tailed *t*-test was used for descriptive statistical analysis (mean ± standard deviation) of quantitative data. A *p*-value of <0.05 was considered to indicate statistical significance.

The university ethics council approved this study (05/02/2013. Decision n° 43). Our study was performed as retrospectively. We have started to do hyperbaric oxygen therapy and ozone therapy on our clinic since 2010 for the patients who had sudden sensorineural hearing loss. We conducted the study in 2013 retrospectively and those patients whom we followed-up and treated from 2010 to 2012 were included in the study. Because of this our ethics board approval was dated 2013.

## Results

### Groups

In total, 106 patients who complied with the participation criteria of the study were selected. The cases in the present study were divided into three groups as follows: 63 patients with oral steroid treatment (Group A), 26 patients with oral steroid + HBOT (Group B), and 17 patients with oral steroid + ozone therapy (Group C). No statistically significant difference was found among the groups regarding age and sex.

### PTA

When the initial pre-treatment PTAs (PTPTAs) of the patients were analyzed, the average of all study participants was 85.8 dB. By group, the values were 80.7 dB in Group A, 92.02 dB in Group B, and 94.9 dB in Group C. No statistically significant difference was observed among the groups (*p* = 0.084) in Kruskal–Wallis tests (Table 1).

**Table 1:** The classification of the initial hearing loss degrees of the groups.

Group	Mild	Moderate	Moderate-severe	Severe	Profound or total
A	3 (4.8%)	10 (15.9%)	15 (23.8%)	14 (22.2%)	21 (33.3%)
B	1 (3.8%)	4 (15.4%)	2 (7.7%)	4 (15.4%)	15 (57.7%)
C	0	3 (17.6%)	2 (11.8%)	2 (11.8%)	10 (58.8%)

A, oral steroid group; B, oral steroid + hyperbaric oxygen group; C, oral steroid + ozone group.

### Response to treatment

When the groups were analyzed using Siegel's criteria according to their recovery levels, Group A showed complete recovery in 14 (22.2%) cases, partial recovery in 10 (15.9%) cases, weak recovery in 8 (12.7%) cases, and no recovery in 31 (49.2%) cases. Group B demonstrated complete recovery in 3 (11.5%) cases, partial recovery in 4 (15.4%) cases, weak recovery in 9 (34.6%) cases, and no recovery in 10 (38.5%) cases. In Group C, complete recovery was observed in 3 (17.6%) cases, partial recovery in 4 (23.5%) cases, weak recovery in 7 (41.2%) cases, and no recovery in 3 (17.6%) cases (Table 2).

**Table 2:** The groups’ response rates to treatment according to siegel's criteria.

Group	Complete recovery	Partial recovery	Weak recovery	No recovery	Total
A	14 (22.2%)	10 (15.9%)	8 (12.7%)	31 (49.2%)	63
B	3 (11.5%)	4 (15.4%)	9 (34.6%)	10 (38.0%)	26
C	3 (17.6%)	4 (23.5%)	7 (41.2%)	3 (17.6%)	17

A, oral steroid group; B, oral steroid + hyperbaric oxygen group; C, oral steroid + ozone group.

A PTA greater than 15 dB was considered a response to treatment, whereas a PTA less than 15 dB was deemed no response to treatment. The highest response rate was found in the oral steroid + ozone group (Group C) (82.3%), followed by the oral steroid + HBOT group (Group B) (61.5%) and the oral steroid group (Group A) (50.8%).

Pearson's chi-squared tests indicated no significant differences in the response to treatment between Groups A and B (*p* < 0.355). Group C showed a significantly higher response rate to treatment than Group A (*p* = 0.019). There were no significant differences between groups B and C (*p* = 0.146) (Table 3).

**Table 3:** The groups’ response to the treatment.

Groups	Response to treatment	No response to treatment	Total
A	32 (50.8%)	31 (49.2%)	63
B	16 (61.5%)	10 (38.5%)	26
C	14 (82.4%)	3 (17.6%)	17

A, oral steroid group; B, oral steroid + hyperbaric oxygen group; C, oral steroid + ozone group.

There is response to treatment: gaining PTA above 15 dB.

There is no response to treatment: gaining PTA less than15 dB.

Regarding SDSs, a statistically significant increase was found following treatment in each of the three groups (*p* = 0.002), with no statistically significant difference in the degree of increase among the groups.

The audiogram curve was upsloping in 16 (15%) patients, downsloping in 32 (30.1%), flat in 25 (23.5%), and indicated total deafness in 33 (31.1%) patients. The groups participating in the study were evaluated as showing a ‘response to treatment’ or ‘no response to treatment’ according to the types of audiograms (Table 4).

**Table 4:** The groups’ response to treatment according to audiogram shape.

Audiogram type	Response to treatment	No response to treatment
*Group A*		
Upsloping	9 (75.0%)	3 (25.0%)
Flat	8 (47.1%)	9 (52.9%)
Downsloping	13 (68.4%)	63 (1.6%)
Total deafness	2 (13.3%)	13 (86.7%)

*Group B*		
Upsloping	3 (75.0%)	1 (25.0%)
Flat	3 (50.0%)	3 (50.0%)
Downsloping	7 (87.5%)	1 (12.5%)
Total deafness	2 (37.5%)	13 (62.5%)

*Group C*		
Upsloping	–	–
Flat	1 (50.0%)	1 (50.0%)
Downsloping	5 (100.0%)	0 (0%)
Total deafness	8 (80%)	2 (20%)

A, oral steroid group; B, oral steroid + hyperbaric oxygen group; C, oral steroid + ozone group.

There is response to treatment: gaining PTA above 15 dB.

There is no response to treatment: gaining PTA less than15 dB.

The efficiency of steroid treatment in patients with profound hearing loss (91 dB and above) was low; adding HBOT or ozone therapy to the treatment significantly contributed to treatment success.

Considering patients with profound hearing loss only, a statistically significant difference in response to treatment was found between Groups A and B (*p* = 0.012) and especially between Groups A and C (*p* = 0.002). No statistically significant difference was found between groups B and C (Fisher's exact test) (Table 5).

**Table 5:** Response to the treatment in patients with profound hearing loss.

Patients with profound hearing loss	Response to treatment	No response to treatment	Total
Group A	4 (19%)	17 (81%)	21
Group B	9 (60%)	6 (40%)	15
Group C	8 (80%)	2 (20%)	10

A, oral steroid group; B, oral steroid + hyperbaric oxygen group; C, oral steroid + ozone group.

## Discussion

The factors affecting prognosis in ISSNHL include the patient's age, existence of vertigo, degree of initial hearing loss, shape of the audiogram, and period between the onset of hearing loss and start of treatment. The degree of hearing loss is an important factor that determines the response to treatment. Profound hearing loss of 90 dB and above indicates a poor prognosis.

Byl et al.^4^ reported an 83% rate of recovery in patients with mild hearing loss and 22% recovery in those with severe hearing loss. In their placebo-controlled study that included steroid treatment, Wilson et al.^9^ observed complete recovery in all of the patients with a hearing loss of 40 dB or less and in patients with a U-shaped hearing loss up to 85 dB. However, only 24% of those with a flat hearing loss of 90 dB and above appeared to recover; in addition, none showed total recovery. Steroid treatment was most effective in patients with a hearing loss of between 40 dB and 90 dB; only 38% of these patients recovered in the placebo group, whereas approximately 78% recovered in the steroid group.

In this study, patients with profound hearing loss showed a lower response to treatment than did those with mild loss. In addition, there was a statistically significant difference between the oral steroid group and the oral steroid + HBOT group (*p* = 0.012; Pearson's Chi-squared test). There was an even greater difference between the oral steroid and oral steroid + ozone groups (*p* = 0.002; Pearson's Chi-squared test).

However, the efficiency of the oral steroid treatment was low in patients with profound hearing loss. In the present study, the response to treatment with a total or close-to-total type of audiogram configuration was low, in concordance with the literature.

Recovery in ISSNHL mostly occurs within the first 2 weeks. The longer the recovery time, the worse the prognosis. Complete or partial recovery without treatment is observed in many patients (32–65%).^9^, ^10^

Presently, more than 60 treatment protocols have been defined, but no precise consensus exists regarding which is best. One widely accepted treatment is steroid treatment.

Steroids have several effects on the inner ear. They suppress the immune system and increase the microcircular current. They have mineralocorticoid effects, and are thought to influence ISSNHL by reducing the endolymphatic pressure. However, the precise underlying mechanism remains unknown.^11^, ^12^

There are two types of corticoid receptors, type 1 and type 2, in cochlear and vestibular cells.^13^, ^14^ When glucocorticoid receptors are activated, a specific gene expression program is launched; thus, the synthesis of inflammatory mediators and cytokines is inhibited, resulting in anti-inflammatory effects.

In the first randomized, controlled study on the use of steroids in ISSNHL, the authors compared a steroid and a placebo. In the active medication group, they administered prednisolone orally in gradually reduced amounts for 10–12 days. All of the patients (*n* = 14) with moderate-frequency hearing losses recovered regardless of the type of treatment. In patients whose loss was worse than 90 dB at all frequencies, no difference could be found in terms of recovery between the steroid and placebo treatment groups. In the remaining patient population (with non-severe hearing loss and hearing at 4 kHz better than at 8 kHz), significant recovery was monitored in the group that was administered steroids. While complete recovery was achieved in 78% of those administered steroids, those administered the placebo achieved 38% partial recovery.^9^ In a previous prospective randomized study, 89% of a group administered corticosteroid treatment recovered, whereas only 44% of the control group administered placebo recovered.^7^

HBOT has been used to treat inner-ear disorders since the early 1970s.^15^ In the late 1970s, several researchers suggested that circulatory disorders were the main reason for ISSNHL. Consequently, HBOT became a comprehensive treatment option for ISSNHL.^16^

The purpose of HBOT in the treatment of ISSNHL is to increase the partial pressure of oxygen in the blood and then, via diffusion, to increase the partial pressure of oxygen in the inner ear fluids that nourish the sensory and neural elements of the cochlea.^17^

Fujimura et al.^18^ claimed that HBOT had a significant additional effect when used in combination with systemic steroid therapy in 43 patients, when compared with 51 patients who were treated with steroids alone. In patients with initial hearing levels of ≥80 dB, the hearing improvement rate was significantly higher in the HBOT group than in the steroid group, whereas in patients with initial hearing levels of <80 dB, the hearing improvement rate was not statistically different between the two groups.

Alimoglu et al.,^19^ who considered 217 patients, administered an oral steroid to 58 patients, oral steroid + HBOT to 61 patients, intratympanic steroid to 43 patients, and only HBOT to 57 patients. The treatment success rate was highest in the steroid + HBOT group (86.88%; 53/61), followed by the oral steroid group (63.79%; 37/58), the intratympanic steroid group (46.51%; 20/43), and the only HBOT group (43.85%; 25/57).

Topuz et al.^20^ showed that HBOT had a greater effect on hearing at low frequencies than at higher frequencies, particularly in young patients. In particular, patients younger than 50 in the HBOT group had better hearing outcomes.

Although the etiology and pathogenesis of ISSNHL are not completely understood, vascular causes are among the primary underlying etiologies. Except for those with a definite contraindication, adding HBOT to the treatment of ISSNHL is a common strategy.

In our study, the group administered steroid treatment experienced a 50.8% treatment response, whereas the steroid + HBOT group had a better response, at 61.5%.

Although the patients who received steroid treatment and those who received HBOT in addition to steroid treatment showed similar responses to treatment, the HBOT supplement positively contributed to the recovery of patients with severe or profound hearing loss.

Ozone therapy is a treatment strategy for ISSNHL because it enhances oxygen, glucose, and ATP delivery to ischemic tissues, and leads to vasodilation, increasing the amount of nitric oxide, stimulating angiogenesis, and providing immunomodulation. Hydrogen peroxide, a derivative of reactive oxygen metabolites that generates ozone in the body, is now widely recognized as an intracellular signaling molecule that activates a tyrosine kinase, which phosphorylates a transcription factor (Nuclear Factor kappaB; NF-κB), which allows the synthesis of several different proteins.^21^, ^22^, ^23^

Recently, a new hypothesis of pathological activation of the cellular stress pathways involving NF-κB in the cochlea was suggested. Pathological activation of NF-κB can result in the production of inflammatory cytokines and other stress-related proteins that can disrupt the homeostatic balance of a cell or tissue.^24^ After ozonation, H_2_O_2_ freely diffuses into the cytoplasm and activates specific protein kinases, which – by phosphorylating I[kappa]B bound to NF-κb – regulate its action and allow migration of the transcription heterodimer p50–p65 into cell nuclei, where it activates gene expression.^25^ Ozone also induces up-regulation of antioxidant enzymes in several cell types, which effectively re-equilibrates the oxidant–antioxidant imbalance.^25^ Therefore, ozone therapy can be an effective method of treatment according to the cellular stress hypothesis in ISSNHL.

In a study by Ragab et al.^26^ comprising 45 patients with ISSNHL at Menoufiya University Hospital (Egypt) between 2004 and 2006, the study group (30 patients) was administered ozone therapy through the autohemotherapy method, while the placebo group (15 patients) received sterile distilled water infusions. Both of the patient populations/groups received 10 session treatments twice a week. The improvement/recovery of hearing in the study group that received the ozone therapy proved to be 77%, whereas the recovery of hearing in the placebo group was 40%, a significant difference. Ozone therapy, by producing reactive oxygen derivatives, causes the hemoglobin-oxygen dissociation curve to slide/slip to the right, thereby causing the oxygen to be more easily delivered into tissues; furthermore, this therapy enhances nitric oxide oscillation/release, which is a powerful vasodilator of endothelial cells, and fixes ischemia in the cochlea. In addition to these features, ozone therapy increases antioxidant efficiency and becomes effective for the treatment of ISSNHL by impacting cellular stress.^26^

In another study, 27 patients with tinnitus were administered ozone therapy, 26 were administered betahistine treatment, and 15 comprised a control group; a significant decline was observed in average tinnitus disability questionnaire scores and subjective tinnitus scores in the ozone group after treatment (*p* < 0.001). A 22.2% recovery rate was achieved according to the severity of tinnitus in patients, a 44.4% recovery rate was achieved according to the tinnitus disability questionnaires, and a 40.7% recovery rate was determined according to the subjective tinnitus scores.^27^

In this study, in the steroid treatment group (Group A), there was complete recovery in 14 (22.2%) cases, partial recovery in 10 (15.9%), weak recovery in 8 (12.7%), and no recovery in 31 (49.2%). In the group administered ozone therapy in addition to steroid treatment (Group C), 3 cases (17.6%) had total recovery, 4 cases had partial recovery (23.5%), and 7 cases had weak recovery (41.2%); however, no recovery was observed in 3 (17.6%) of the cases in this group. The highest response rate to treatment was in the steroid + ozone therapy group (Group C) (82.4%), followed by the steroid + HBOT group (Group B) (61.5%), and oral steroid group (Group A) (50.8%).

## Conclusion

The results of our study demonstrate that ozone therapy significantly contributes to the recovery process in treatment of ISSNHL; however, we believe that further placebo-controlled studies should be performed with a greater number of patients receiving ozone therapy for a longer period and with a higher ozone dose.

## Conflicts of interest

The authors declare no conflicts of interest.
